# Le tabagisme chez les patients vivant avec le VIH (PvVIH) suivis au Centre de Traitement Ambulatoire de l'Hôpital Fann de Dakar

**DOI:** 10.11604/pamj.2019.34.42.14526

**Published:** 2019-09-20

**Authors:** Fatimata Binetou Rassoule Mbaye, Nafissatou Oumar Toure, Ndeye Fatou Ngom Gueye, Malick Kane, Kiné Ndiaye, Yacine Dia Kane, Ulrich Davy Kombila, Cheikh Tiidiane Ndour, Khady Thiam, Maïmouna Fafa Cisse, El Hadji Mamadou Ndiaye

**Affiliations:** 1 Service de Pneumologie, Centre Hospitalier National Universitaire de FANN (CHNUF), Dakar, Sénégal; 2 Centre de Traitement Ambulatoire, Centre Hospitalier National Universitaire de FANN (CHNUF), Dakar, Sénégal; 3 Service de Médecine Interne, Centre Hospitalier Universitaire de Libreville (CHUL), Libreville, Gabon; 4 Service des Maladies Infectieuses - Centre Hospitalier National Universitaire de FANN (CHNUF), Dakar, Sénégal; 5 Service de Pneumologie, UFR Santé de Thiès, Dakar, Sénégal

**Keywords:** Tabagisme, PvVIH, facteurs de risque cardiovasculaires, Smoking, PLHA, factors of risks of cardio-vascular diseases

## Abstract

**Introduction:**

Le tabagisme doublerait la mortalité chez les PvVIH un risque accru de survenue de maladies non classant Sida. La prévalence de la consommation de tabac est plus élevée chez les PvVIH que dans la population générale. Nous nous sommes fixés comme objectifs d'évaluer la prévalence du tabagisme chez les PvVIH, de décrire les caractéristiques cliniques et spirométriques des fumeurs et ex-fumeurs et d'évaluer leurs connaissances et attitudes face au tabagisme.

**Méthodes:**

Nous avons donc mené une étude transversale, descriptive et analytique du 15 Juillet au 15 Décembre 2015, chez les PvVIH suivis au Centre de Traitement Ambulatoire du Centre Hospitalier Universitaire National de Fann.

**Résultats:**

La population d'étude concernait trois cents (300) PvVIH. Le sex-ratio était de 0,8. Nous avons retrouvé 15% de fumeurs et 23,7% d'ex-fumeurs. L'âge moyen des fumeurs était de 44,38 ± 9,55 ans. La quasi-totalité des fumeurs 91,1% avait déjà commencé à fumer avant la connaissance de leur statut sérologique et 35,6% d'entre eux avaient majoré leur consommation de tabac après. Les signes respiratoires étaient dominés par la gêne respiratoire dans 64,4% des cas chez nos fumeurs. Parmi les fumeurs qui ont bénéficié de la spirométrie, 67% d'entre eux avait un trouble ventilatoire obstructif non amélioré par les béta2-mimétiques et 28,1% un syndrome restrictif. Parmi les ex-fumeurs, 40,8% affirmait que le statut sérologique était le motif de sevrage.

**Conclusion:**

Le tabagisme peut être initié ou majoré après connaissance du statut sérologique. Il peut être à l'origine de nombreuses complications chez le PvVIH.

## Abstract (en)

**Introduction:**

PLHA who smoke have twice the never-smoker mortality rate and have an increased risk of developing non-AIDS diseases. The prevalence of tobacco smoking is higher among PLHA than in the general population. The purpose of this study was to assess the prevalence of smoking among PLHA, to describe the clinical and spirometric features of smokers and ex-smokers and to assess their knowledge and attitudes toward smoking.

**Methods:**

We conducted a cross-sectional, descriptive and analytical study among PLHA followed up in the Outpatient Department of the National University Hospital Center of Fann from 15 July to 15 December 2015.

**Results:**

Three hundred (300) PLHA were included in the study. Sex ratio was 0.8. Out of the study population, 15% were smokers and 23.7% were ex-smokers. The average age of patients was 44.38±9.55 years. The quasi-totality of the smokers (91.1%) had already started smoking before the detection of the serological status and 35.6% of them had increased tobacco use after. Respiratory symptoms among smokers were dominated by respiratory distress (64.4%). Smokers who underwent spirometry had obstructive ventilatory impairment not improved by beta-2-mimetic agents (67%) and restrictive disease (28.1%). Out of ex-smokers, 40.8% reported that their serological status was the reason for smoking cessation.

**Conclusion:**

**P**eople may begin or increase smoking after knowledge of serological status. In PLHA, smoking causes cardiovascular and respiratory diseases as well as complications.

## Introduction

Le tabac est la première cause de mortalité évitable dans le monde. Sa consommation est l'une des plus graves menaces qui pèse sur la santé publique mondiale. Elle tue près de 7 millions de personnes chaque année. On estime que le tabac tue jusqu'à un sur deux de ses consommateurs. Aucun autre facteur de risque n’est associé à un taux de mortalité aussi élevé et ne cause plus de 500 milliards de dollars par an de dommages économiques [1]. La consommation de tabac diminuant dans les pays à revenus élevés, l’industrie du tabac se tourne progressivement vers les pays à revenus moyens et faibles, particulièrement en Afrique, en Asie et en Europe de l’Est, pour attirer de nouveaux consommateurs. En l'absence de politiques complètes de prévention et de lutte antitabac, on estime que la prévalence du tabagisme va augmenter en Afrique de près de 39% à l'horizon 2030. Plus de 5 millions d'entre eux sont des consommateurs ou d'anciens consommateurs, et plus de 600.000 des non-fumeurs involontairement exposés à la fumée [1]. Le tabagisme n'épargne pas non plus les patients vivant avec le VIH qui déjà sont fragilisés par leur statut, au contraire le tabagisme doublerait la mortalité chez cette population avec un risque accru de survenue de maladies non classant Sida. Nous avons effectué cette étude dans le but: 1) d'étudier la prévalence du tabagisme au sein des PvVIH suivis au CTA du CHUN de Fann; 2) de déterminer les connaissances, attitudes et comportement des PvVIH face au tabagisme.

## Méthodes

**Type et cadre d'étude:** il s'agissait d'une étude transversale à visée descriptive et analytique effectuée chez des PvVIH suivis au CTA du Centre Hospitalier Universitaire de Fann (CHNU). L'enquête s'est déroulée sur 05 mois: entre le 15 juillet et le 15 décembre 2015.

**Recueil de données:** le recueil des données s'est fait sur la base d'un questionnaire auto-administré standardisé, anonyme (annexe), destiné aux PvVIH suivis au centre de traitement ambulatoire de Fann. Des explications ont été données individuellement à chaque patient par l'enquêteur, sur les objectifs de l'enquête, les modalités de remplissage, tout en insistant sur l'anonymat. Le questionnaire comprenait des questions ouvertes et à choix précis et concernait: 1) le statut démographique et le niveau socio-économique des patients: âge, sexe, niveau d'étude, profession et revenu mensuel des patients; 2) le statut sérologique des PvVIH: date et circonstances du diagnostic, profil sérologique, infections opportunistes, stade OMS au début et au moment de l'étude, CD4 et charge virale au début et à l'inclusion, traitement ARV durée et protocole; 3) le statut tabagique du fumeur: âge de début, durée tabagisme, motif d'entrée, facteurs influençant le tabagisme, dépendance selon le test de Fagerstrom, autres addictions, souhaits de sevrage tentatives d'arrêts et motifs; 4) attitudes des ex-fumeurs: durée d'arrêt, motif d'arrêt, lien avec le statut sérologique; 5) quel que soit le statut tabagique: connaissance des effets respiratoires du tabagisme actif et passif et autres méfaits, l'estimation personnelle du niveau d'information concernant le tabagisme (informations fournies au CTA, par les médiats, etc..).

**Critères d'inclusion:** tous les patients VIH suivis au CTA qui ont bien voulu participer à l'étude et répondre au questionnaire.

**Critères d'exclusion:** les patients qui ont refusé de répondre au questionnaire quel que soit leur raison.

**Déroulement de l'enquête:** le consentement verbal et éclairé du patient était obtenu avant qu'il ne remplisse lui-même le questionnaire ou par le médecin investigateur s'il était illettré. Une consultation d'aide au sevrage tabagique avait été ensuite proposée aux fumeurs.

**Saisie et analyse des données:** la saisie des données a été réalisée à l'aide du logiciel Epi Info 7. Le traitement des données était fait par le statisticien avec le logiciel SPSS 21. Les tableaux de fréquence avaient permis l'analyse des données uni-variées. Le test de Chi2 était utilisé pour l'analyse bi-variée pour trouver le lien entre les variables qualitatives. Le test Anova était utilisé pour l'analyse bi-variée pour trouver le lien entre les variables quantitatives. Le seuil de validité pour tous ces tests était arrêté à p< 0,05.

**Considération éthique:** le respect de la dignité du PvVIH répondant aux questions a été observé. La confidentialité entre le patient et l'investigateur a également été observée par l'administration d'un questionnaire anonyme.

## Résultats

### Caractéristiques de la population générale

**Données socio-démographiques:** l'étude a porté sur 300 PvVIH dont 45 fumeurs soit 15% avec un sex-ratio de 0,81. L'âge moyen était de 44,38 ans avec des extrêmes de 27 et 64 ans. Plus de la moitié des patients c’est-à-dire 64,6% (n=194) avait un âge compris 35 et 54 ans. Les femmes étaient plus représentatives pour les tranches [15-24], [25-34] et [35-44] avec respectivement 66,7%, 58,7% et 67% (p=0.035). Notre population d'étude se composait de 137 mariés (45,7%), 63 célibataires (21,0%), 56 veufs (18,7%) et 44 divorcés (14,7%) (p=0,039).

**Antécédents et comorbidités:** outre le tabac, plus d'¼ soit 26,7% des patients présentaient d'autres facteurs de risque cardio-vasculaires tels que: le surpoids (IMC = 24,5 kg/m^2^) présent chez 31,7% (n=95); l'hypercholestérolémie totale, retrouvée à 10,6%, le diabète 4%, et l' HTA à 18%. La co-infection TB-VIH était présente chez 34 patients soit 11,3% de la population étudiée au moment du diagnostic. L'hépatite virale B était présente chez 22 patients soit (7,3%). La co-infection VIH/TB/AgHbs était retrouvée chez un seul patient.

**Répartition selon le statut tabagique:** nous avons enregistré 15% (n=45) de fumeurs, 71 ex-fumeurs soit 23,7% et 61,3% non-fumeurs soit 184 patients.

**Figure 1:**
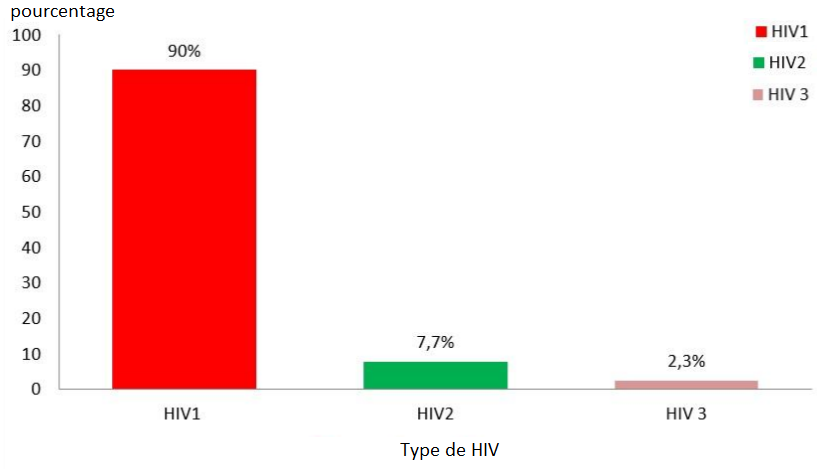
Répartition de la population selon type de VIH

**Répartition selon le statut sérologique:** la grande majorité (90%) (n=270) de la population était infectée par le VIH1, le VIH2 regroupait 7,70% des cas (n=23) et les patients double-profil (VIH1+VIH2) étaient les moins représentés avec 2,3% (n=7) (Figure 1). Au moment du diagnostic de l'infection à VIH, le stade III OMS était plus représentatif avec 39,0% (n=117), suivi respectivement du stade I avec 27,3% (n=82), du stade II (23%) (n=69) et les 10,7% restants (n=32) étaient classés au stade IV par l'OMS. Plus de la moitié des patients 58% (n=174) présentait au moins une infection opportuniste. La majorité des PvVIH 83% (n=249) était sous traitement ARV (antirétroviral) dont en première ligne et 9% (n=27) en seconde ligne. Les 8% restant soit 24 étaient naïfs d'ARV.

**Caractéristiques des fumeurs:** on retrouvait une nette prédominance masculine avec un sex-ratio de 3,09. Le profil VIH 1 était largement retrouvé dans 93,3% des cas (n=42). Près d'un quart soit 22,7% (n=11) des fumeurs avaient un taux de CD4 inférieur à 350 éléments/mL et 86,7% (n=39) avait une copie virale inférieure à 1000 copies/ml. La majorité des fumeurs soit 95,6% (n=43) était déjà sous traitement ARV.

La quasi-totalité des fumeurs soit 97,7% (n=44) utilisait les cigarettes manufacturées; seul un patient fumait du tabac noir roulé traditionnel (appelé poon en langue nationale wolof). Près d'un tiers des fumeurs soit 28,9% (n=13) présentait une intoxication alcoolo-tabagique et 8,9% d'entre eux (n=4) faisaient aussi usage du cannabis. Cependant ils présentaient presque tous une dépendance moyenne à légère avec respectivement 68,9% (n=31) et 28,90% (n=13).

La majorité des fumeurs 84,5% (n= 40) avait un tabagisme ancien supérieur à 10 ans contre 11,1% (n=5) qui avait fumé moins de 10 ans. Seuls 8,90% (n=4) ont commencé à fumer après connaissance de leur statut sérologique, mais 35,6% des fumeurs (n=16) affirmaient que la connaissance de leur statut sérologique avait augmenté leur consommation en tabac.

Les facteurs favorisant le tabagisme de nos PvVIH étaient la solitude (33,30%), le stress 20%), certaines circonstances telles que les festivités entre amis (15,50%). Les signes respiratoires les plus fréquemment retrouvés chez nos fumeurs étaient la gêne respiratoire dans 64,4% des cas (n=29) et une douleur thoracique dans 37,8% des cas (n=17).

Quarante pour cent (40%) des fumeurs (n=120) avaient des antécédents de tuberculose quelle que soit sa localisation (pulmonaire, pleurale, ganglionnaire, péritonéale). Seule la moitié des patients ont pu bénéficier de la spirométrie et parmi eux 67% (n=13) avait un TVO (trouble ventilatoire obstructif) modéré avec une moyenne de VEMS (Volume Expiratoire Maximal par Seconde) à 59% de la théorique non amélioré par les b2-mimétiques et 28,1% (n=6) avait un syndrome restrictif.

**Figure 2:**
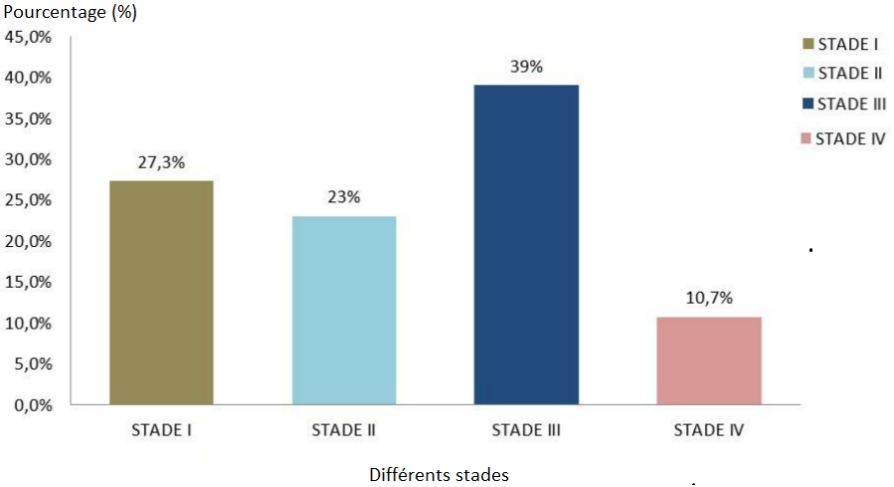
Répartition des fumeurs selon la dépendance

Le sevrage tabagique était proposé aux fumeurs et 89,9% d'entre eux (n=40) affirmait vouloir arrêter de fumer et parmi eux, 51,1% (n=23) affirmaient avoir besoin à la fois d'une aide psychologique et médicamenteuse (Figure 2).

### Caractéristiques des ex-fumeurs

L'âge moyen des ex-fumeurs est de 48,68 ans avec des extrêmes allant de 26 et 74 ans avec une prédominance masculine. La majorité des ex-fumeurs soit 88,7% (n=63) était de profil 1, tandis que le profil 2 représentait 11,3% (n=8). Il n'y avait pas de double profil chez les ex-fumeurs (p= 0,336). Comme chez les fumeurs, près des ¾ des ex-fumeurs soit 73,2% (n= 52), quel que soit le profil sérologique, avaient un taux de CD4 égal à 350 éléments/mL et 87,3% (n=62) avait une copie virale < 1000/mL et ils étaient presque tous soit 87,3% (n=62) sous ARV. On retrouvait des antécédents de tuberculose chez 42,3% d'entre eux (n=30). La spirométrie était revenue normale chez 42,9% des ex-fumeurs. La culpabilisation dans (43,7%) et la connaissance de leur statut sérologique dans (40,8%) étaient les principaux motifs d'arrêt du tabac.

### Attitudes et connaissances du tabagisme par la population générale

Plus de la moitié des PvVIH soit 55,3% (n=166) n'avait jamais fumé ou tenté de fumer. Par rapport aux pathologies liées au tabac, 98,3% (n=295) estimaient que le tabagisme est pourvoyeur de pathologies respiratoires en particulier la tuberculose pulmonaire et le cancer du poumon. Les pathologies extra-respiratoires étaient évoquées par 70,7% des patients (n=212) parmi lesquelles l'ulcère gastrique, l'infarctus du myocarde et l'hypertension artérielle. La quasi-totalité des PvVIH (90,3% n=298), surtout les ex-fumeurs et non-fumeurs (100% chacun), étaient conscients des dangers du tabagisme passif. Cinquante et un pour cent (51%) des PvVIH (n=123), avaient reçu des séances de communication pour le changement de comportement pendant les groupes de parole par rapport au tabagisme, cependant la presque totalité des PvVIH soit 99% (n=297), quel que soit le comportement tabagique, trouvait nécessaire de disposer d'une bonne information sur les méfaits du tabac.

## Discussion

### Caractéristiques socio-démographiques et cliniques de la population d'étude

Notre échantillon englobait 300 patients vivant avec le VIH. La moyenne d'âge de notre population d'étude de 44,8 ans était superposable aux 45,1 ans de Duval *et al.* [2] et aux 42,4 ans de Coulibaly [3]. On retrouvait une prédominance féminine avec un sex-ratio de 0,81. Les autres études africaines, en particulier au Sénégal par Coulibaly [3], au Mali par Traoré [4] et en Guinée Conakry par Loua [5] corroboraient nos résultats avec respectivement un sex-ratio de 0,7, 0,75 et 0,5. Cette féminisation de l'infection à VIH dans nos régions s'explique pour plusieurs raisons: 1) Par une vulnérabilité anatomique du fait que la muqueuse génitale féminine est souvent porteuse de microtraumatismes et joue le rôle de réceptacle favorisant un contact prolongé avec le sperme potentiellement infecté. 2) Elle est aussi d'ordre socio-économique du fait de la dépendance financière de la gente féminine par rapport à leur conjoint, dans les pays en voie de développement et du poids de la société traditionnelle. 3) Mais d'autre part, peut-être, par le fait que les femmes se font dépistées plus que les hommes (ex: dépistage systématique pendant la grossesse).

D'après ONUSIDA 2007, il existe une féminisation de l'épidémie du VIH surtout en Afrique subsaharienne où les femmes représentent 61% de tous les adultes vivant avec le VIH [6]. De plus au Sénégal, la dernière enquête démographique compte 02 femmes infectées par le VIH pour 1 homme [7]. La plupart de nos patients étaient mariés 45,7% (n=137). Ce constat, partagé par d'autres études dont celle de Coulibaly [8] qui retrouvait 55,53% de mariés, ce qui confirme que l'infection à VIH est désormais un problème de famille. En effet, depuis l'ère de la trithérapie gage d'un meilleur contrôle de l'infection avec charge virale indétectable, de la systématisation de la prévention de la transmission mère-enfant (PTME) et de la prise en charge psycho-sociale (éducation sur la santé de la reproduction et la planification familiale), nous constatons de plus en plus d'unions conjugales entre les PvVIH.

Nous avions retrouvé d'autres facteurs de risque cardio-vasculaires autres que le tabac tels que le surpoids (IMC = 24,5 kg/m^2^) à 26,92% et l'hypercholestérolémie à 10,6% étaient retrouvés à des proportions inférieures à ceux de Ndiaye [9] avec 33,67%. Nous dénombrions aussi 4% de diabétiques, prévalence supérieure à celle Aw [10] de l'ordre de 2,3%. L'hypertension artérielle à 18% était assez proche des 14,7% retrouvés par Aw [10]. Ces facteurs de risque cardio-vasculaires constituent avec le tabagisme les principales comorbidités retrouvées chez les PvVIH depuis l'avènement de la trithérapie ARV. En effet les ARV peuvent induire des perturbations métaboliques comme les dyslipidémies, insulino-résistance entre autres.

### Caractéristiques sérologiques de la population d'étude

Le sérotype VIH 1 était majoritairement retrouvé à 90%, taux superposable à celui de Aw [10], Coulibaly [3] et Diène [11] respectivement 91,5%; 92% et 94,32% au Sénégal et ceux de Loua [5] avec 98,6% à Conakry. Cette nette prédominance du VIH 1 s'explique par sa plus grande virulence, qui se transmet plus facilement aussi bien par voie sexuelle, que de la mère à l'enfant. De même, sa durée d'incubation est beaucoup plus courte qu'en cas d'infection par le VIH 2. Dans notre étude, le taux moyen de CD4 à l'inclusion au TARV était de 227 avec des extrêmes de 1 et 698 éléments/ml. Nos résultats étaient supérieurs à ceux retrouvés par Diène [11] et Ba [12] avec respectivement 157 CD4/mL et 189 CD4/mL, par contre inférieurs à ceux d'Arnaud [13] et Ndiaye [9] avec respectivement 295 CD4/mL et 272 CD4/mL. Dans la cohorte ANRS CO3 Aquitaine, Bernard [14] retrouvait 59,8% de patients ayant un taux de CD4 égal à 350/mL, contre 21,70% (n=65) dans notre population d'étude.

Ces taux bas de CD4 témoignant d'une immunodépression sévère est une conséquence directe du retard diagnostique en Afrique. Jarousse *et al.* en France [15] rapportent qu'à l'image de ce qui se passe en Afrique, l'infection est diagnostiquée plus tardivement chez les africains vivants en France par rapport aux patients d'origine française. Cependant, en cours d'évolution cette médiane était remontée à 515/mL ce qui est largement supérieure à celle des résultats de Aw [10] avec une médiane à 413/mL. Cette évolution du taux de CD4 est imputable au suivi régulier chez nos patients et à l'efficacité de la tri-thérapie antirétrovirale. En effet les ARV agissent par divers mécanismes pour bloquer la réplication virale.

### Caractéristiques des fumeurs

Ils représentaient 15% de la population avec une prédominance masculine 75,6% contre 24,4% de femmes. Ces résultats étaient largement inférieurs à ceux des études de Hellerberg [16], de Laure *et al.* [17] de Duval *et al.* [2] dans l'étude ANRS (Agence publique française de Recherches sur le Sida et les Hépatites virales) Vespa2 et de Bernard [14] avec respectivement 60%, 37,5%, 43% avec 51% de fumeurs. Cette différence pourrait s'expliquer par le faible pourcentage de notre population d'étude mais aussi par la faible prévalence du tabagisme en Afrique Sub-saharienne par rapport aux continents Européen et Nord-Américain. En effet Duval *et al.* [2] avaient trouvé une différence de prévalence du tabagisme entre les différentes origines géographiques des PvVIH avec une prévalence de 25% parmi les migrants provenant de l'Afrique sub-saharienne contre 43% pour les Nord-américains et 52% pour les Européens. Mais également cette différence de prévalence pourrait s'expliquer aussi par le fait qu'il y ait dans les autres études une proportion assez importante des usagers de drogues intraveineuse et chez les hommes ayant des rapports avec des hommes (MSM) qui sont plus susceptibles d'être fumeurs quel que soit le statut sérologique [18].

Des études menées dans plusieurs pays ont montré de façon constante que la prévalence du tabagisme chez les membres de la communauté lesbienne et chez les PvVIH/SIDA était plus élevée que dans la population générale [19, 20]. Au Sénégal, la prévalence du tabagisme chez la population adulte en 2015 était de 6% selon l'enquête GATS (Global Adults Tobacco Survey) [21]. Si la plupart de nos fumeurs (91%) (n=40) avait commencé à fumer avant leur diagnostic sérologique, 35,6% (n=16) d'entre eux avaient augmenté leur tabagisme après connaissance de leur statut sérologique. Ceci pourrait laisser croire que le VIH pouvait être un facteur influençant l'intensité du tabagisme chez les fumeurs. Presque tous nos fumeurs (97,7%) utilisaient les cigarettes industrielles. Ce résultat rejoint celui de l'étude de Chaimea [22] où tous les fumeurs utilisaient la cigarette industrielle. Ceci pourrait s'expliquer par l'accessibilité de la cigarette industrielle aussi bien géographique que financière mais surtout par la contrebande initiée et entretenue par les firmes du tabac au niveau de nos frontières.

La quasi-totalité des fumeurs soit 95,6% était sous ARV, taux supérieur aux 80% retrouvés par Duval *et al.* [2]. La prévalence du tabagisme varie avec la durée du traitement ARV, elle est plus importante après 6 ans de traitement ARV. Bernard [14] avait fait aussi la même constatation. En effet depuis l'avènement de la trithérapie, l'espérance de vie des PvVIH a véritablement augmenté, avec baisse importante de la morbi-mortalité liée à la maladie, ce qui inciterait donc ces derniers à se comporter comme les séronégatifs et à user davantage du tabac. Une étude menée chez les PvVIH a montré que les fumeurs perdaient en moyenne 12,3 ans de vie à cause du tabagisme [23]. Grace aux traitements antirétroviraux, les PvVIH ont aujourd'hui une espérance de vie équivalente à celle de la population générale. Il serait dommage de laisser partir ces années en fumée.

Dans notre étude les patients ayant un bon contrôle de l'infection à VIH (CD4 = 350/mL) étaient beaucoup plus susceptibles d'être fumeurs (77,2%) que les patients avec un taux de CD4 < 350/mL (22,70%). Duval *et al.* [2] avait fait les mêmes constatations et avait retrouvé une prévalence de 47% de fumeurs chez les PvVIH ayant un meilleur contrôle de l'infection avec un taux de CD4 > à 500/mL, contre 41% avec des taux de CD4 entre [200-500]. Ces résultats divergeaient avec ceux de Bernard [14] pour qui, les patients avec un meilleur contrôle de l'infection (CD4>350/mm^3^) étaient moins susceptibles d'être fumeurs réguliers (48%) que les patients avec un moins bon contrôle (53%).

Plus de la moitié de notre population d'étude fumait quand ils étaient seuls ou stressés avec respectivement 33,3% et 20%. La conduite addictive le plus souvent associée au tabagisme était le cannabis à 8,9% et l'alcool à 28,9% Duval *et al.* [2] corroborait nos résultats avec des pourcentages plus élevées: 25% d'alcool et 50% de cannabis. Ces résultats inquiètent d'autant plus que l'alcool et le cannabis multiplient les effets du tabac. Nous n'avions pas retrouvé d'UDI dans notre population d'étude contre 12% et 13,4% d'UDI (Usagers de drogues intraveineuses) respectivement chez Duval *et al.* [2] et Laure *et al.* [17].

Plus de la moitié des fumeurs présentait au moins une symptomatologie respiratoire, une gêne respiratoire dans 64,4% des cas et une douleur thoracique dans 37,8%. Près du 1/3 des fumeurs (n=14) avait une dépendance tabagique modérée, Bernard [14] et Duval *et al.* [2] avaient quant à eux retrouvé respectivement 60% et 56% de dépendance modérée et forte. Ceci pourrait s'expliquer par le fait que ces plus dépendants au tabac avaient un moins bon contrôle de l'infection avec un taux de CD4 <350 éléments/ml chez 80% d'entre eux (n=11).

La proportion de fumeurs motivés au sevrage tabagique était de 89,9% quelle que soit la dépendance, 77,8% avaient essayé d'arrêter une fois au moins et 71,1% pensaient avoir besoin d'aide au sevrage tabagique. Ce taux de motivation au sevrage tabagique était largement supérieur à ceux de Duval *et al.* [2] et Bernard [14] avec respectivement 37% et 40% de motivation. Les EFR (Exploration Fonctionnelle Respiratoire) réalisées chez nos fumeurs étaient anormales dans 85,7% des cas chez les gros fumeurs avec un nombre de paquet année (P.A = 10) contre 60% pour ceux qui fumaient moins (P.A<10). Elles étaient d'autant plus anormales avec ceux ayant un moins contrôle de l'infection à VIH CD4 < 350 dans 75% des cas contre 69,2% de patients avec CD4 = 350 éléments/mL). Ceci pourrait être expliqué par le rôle que pourrait avoir l'infection à VIH dans la survenue d'un emphysème pulmonaire, indépendamment du tabagisme [24].

En effet, l'infection par le VIH est un facteur de risque indépendant d'emphysème [25]. Il existe chez les patients VIH un développement plus rapide des lésions emphysémateuses que chez le sujet non VIH. Les hypothèses évoquées sont que l'infection par le VIH en lui-même, ou des infections virales latentes peuvent aggraver ou potentialiser les lésions liées au VIH [26]. Une autre hypothèse serait que le VIH comme le tabac aggrave la colonisation bronchique, ce qui augmente les processus inflammatoires dans le poumon [27].

### Caractéristiques des ex-fumeurs

Notre étude retrouvait 71 ex-fumeurs soit 23,7%. Ce chiffre est proche aux 22,1% obtenus par Laure *et al.* [17] et légèrement supérieur au 17% de Duval *et al.* [2]. L'âge moyen des ex-fumeurs était de 48,68 avec une prédominance masculine pour toutes les tranches d'âge. Les mariés étaient beaucoup plus représentatifs avec plus de la moitié des ex-fumeurs (52,1%). Ceci pourrait être expliqué par l'indisposition de l'entourage avancée par 43,7% des ex-fumeurs comme motif de sevrage. La grande majorité (85,9%) des ex-fumeurs avait arrêté après connaissance de leur statut sérologique, mais cette dernière était un motif de sevrage pour 40,8% d'ex-fumeurs. La connaissance du statut sérologique semblait beaucoup plus être un facteur de sevrage qu'un facteur d'initiation au tabagisme. Comme chez nos fumeurs, la majorité des ex-fumeurs (72,2%) avaient un taux de CD4 ≥ à 350 éléments/mL et 87,3% avaient une CV < à 1000 copies/mL. Ces taux de CD4 obtenus et de copie virale chez les ex-fumeurs semblent être bien corrélés avec le traitement ARV. En effet ils étaient presque tous soit 87,3% sous traitement ARV au moment de l'inclusion.

Ils présentaient des EFR normales dans 42,9% des cas contre 29,4% chez les fumeurs. Ces résultats démontrent l'effet bénéfique du sevrage tabagique sur la fonction respiratoire. D'autres études [28,29] ont montré aussi que l'arrêt du tabac faisait baisser le risque de cancer de 30%, le risque de décès de près de 16% si l'on considère toutes les causes possibles, de 20 à 60% le risque de maladie cardiovasculaire.

### Attitudes et connaissances du tabagisme par la population générale

Nous avions trouvé au sein de notre population d'étude 61,3% de non-fumeurs, taux supérieur à ceux de Duval *et al.* [2] et de Laure *et al.* [17] avec respectivement 40% et 40,4% de non-fumeurs. Ces chiffres concordent au fait que la prévalence du tabagisme était variable selon l'origine géographique des PvVIH et éventuellement à d'autres groupes (les UDI, les MSM entre autres). Cependant la quasi-totalité (99,7%) de la population d'étude avait conscience de la nocivité du tabac que ce soit au niveau respiratoire (98,3%) qu'extra-respiratoire (70,7%). Cette prise de conscience collective pourrait provenir des séances de groupes de parole au niveau du CTA. En effet la moitié des patients (51%) avaient reçu des séances de communication pour le changement de comportement pendant les groupes de parole. Et pendant ces groupes de parole, des informations sur les méfaits du tabac étaient fournies aux PvVIH, sur l'intérêt et les bénéfices du sevrage tabagique, mais surtout le rôle de la prévention. La loi sur l'interdiction de fumer dans les lieux publics était connue par 68,3% de notre population d'étude, son application était souhaitée par la totalité des sujets interrogés. La création de structures d'aide au sevrage, un contact par un professionnel de santé et une campagne nationale étaient cités comme étant les meilleurs moyens pour prévenir et/ou arrêter le tabagisme avec respectivement 95,3%, 89,2% et 89,9% (p=0,053).

## Conclusion

La prévalence du tabagisme est beaucoup plus élevée chez les PvVIH que dans la population générale augmentant les risques de morbidité et de mortalité chez cette population vulnérable. Il est donc primordial que des mesures de prise en charge surtout préventive doivent être mises en place chez cette population vulnérable.

### Etat des connaissances actuelles sur le sujet

- Connaissances des conséquences physiopathologiques, cliniques et thérapeutiques du tabagisme sur le VIH et de la difficulté du sevrage tabagique.

### Contribution de notre étude à la connaissance

- Notre étude a permis de déterminer la prévalence du tabagisme chez les PVVIH suivis au CTA de l'Hôpital Fann de Dakar;
- De montrer que la connaissance du statut sérologique est un facteur aggravant le tabagisme chez nos patients;
- D'insister auprès des autorités sur la nécessité de la prévention du tabagisme chez les PvVIH.

## Conflits d’intérêts

Les auteurs ne déclarent aucun conflit d'intérêts.

## References

[1] ONUSIDA Vue d'ensemble de l'épidémie de sida - 2014.

[2] Duval X, Gabriel B, Daniel G, Villes V, Dupré T, Leport C (2008). Living with HIV, antiretroviral treatment experience and tobacco smoking: results from a multisite cross-sectional study. Antivir Ther.

[3] Coulibaly JC Les affections neuro-méningées au cours de l'infection à VIH à la clinique des maladies infectieuses du CHNU de Fann: prévalence et facteurs associés au décès.

[4] Traoré MS (2008). Etude bibliographique des thèses réalisées sur le VIH/SIDA de Janvier 2005 à Décembre 2006.

[5] André Loua, Cécé Dominique Dramou, Nyankoye Yves Haba, Fodé Bangaly Magassouba, Mathieu Lamah, Abdoulaye Camara (2017). Profil hématologique des patients infectés par le VIH à Conakry: cas anatomo-clinique. Hématologie.

[6] OMS Le point sur l'épidémie de sida 2007.

[7] Oulibaly D (2006). Les causes liées aux décès des patients sous traitement antirétroviral au Service des Maladies Infectieuses et Tropicales de l'Hôpital du Point G.

[8] Coulibaly AM (2012). Mortalité sous trithérapie ARV: prévalence et facteurs associés chez les patients infectés par le VIH sous traitement ARV de 1ere ligne suivis au centre de traitement ambulatoire du CHNU de Fann de Dakar. Thèse Med Dakar.

[9] Ndiaye O (2015). Etude des troubles lipidiques et autres facteurs de risque cardio-vasculaires chez les personnes vivants avec le VIH sous ARV: expérience de l'hôpital régional de Thiès. Thèse Med Dakar.

[10] Aw F (2015). Les facteurs de risque cardiovasculaires chez les personnes vivants avec le VIH: étude transversale multicentrique à propos de 133 cas. Thèse Med Dakar.

[11] Diène A (2015). Les anomalies de l'hémogramme et leur fréquence chez les sujets infectés par le VIH au cours du traitement ARV au CTA de Fann. Thèse Med Dakar.

[12] Ba K (2011). Décentralisation des activités de la prise en charge des patients vivants avec le VIH au Sénégal: expérience du district sanitaire de Linguère (Louga). Thèse Med Dakar.

[13] Arnaud VAR (2010). Bilan de 10 années d'activités de prise en charge de l'infection à VIH au Centre De Promotion de la Santé Cardinal Hyacinthe Thiamdoum (CPS/CHT) de Dakar (1999-2009). Thèse Med Dakar.

[14] Bernard A, Tessier JF, Rambeloarisoa J, Bonnet F, Fossoux H, Neau D (2006). HIV infection and tobacco smoking behaviour: prospects for prevention? ANRS CO3 Aquitaine Cohort, 2002. Int J Tuberc Lung Dis.

[15] Jarrousse B, Deze C, Akonde A, Lurton G (2011). Prise en charge clinique de l'infection VIH dans les pays à ressources limitées.

[16] Helleberg M, Margaret May T, Suzanne Ingle M, Dabis Francois, Reiss Peter, Fätkenheuer Gerd (2015). Smoking and life expectancy among HIV-infected individuals on antiretroviral therapy in Europe and North America. the ART Cohort Collaboration.

[17] Tron Laure, Lert France, Spire Bruno, Dray-Spira Rosemary, the ARNS-Vespa2 study group (2014). Tobacco Smoking in HIV-Infected versus General Population in France: Heterogeneity across the Various Groups of People Living with HIV. PLoS One.

[18] OMS Le point sur l'épidémie de sida 2007.

[19] Rath Jessica M, Villanti Andrea C, Rebecca Rubenstein A, Vallone Donna M (2013). Tobacco use by sexual identity amoung adults in the United States. Nicotine & Tobacco Research.

[20] Hongying Dai (2017). Tobacco product use amoung lesbian, gay and bisexual adolescents. Pediatrics.

[21] Agence Nationale de la Statistique et de la Démographie (ANSD) et Ministère de la Santé et de l'Action Sociale (2015). Enquête mondiale sur le tabagisme chez les Adultes (Global Adults Tobacco Survey, GATS)- Sénégal.

[22] Helleberg M, May MT, Ingle SM, Dabis F, Reiss P, Fätkenheuer G (2015). Smoking and life expectancy among HIV-infected individuals on antiretroviral therapy in Europe and North America. AIDS.

[23] Chaimea M (2013). Le tabagisme chez lez étudiants en médecine à propos d'une enquête menée auprès de 500 étudiants de l'UCAD. Thèse Med Dakar.

[24] Petrache I, Diab K, Knox KS, Twigg HL, Stephen RS, Flores S (2008). HIV-associated pulmonary emphysema: a review of the literature and inquiry into its mechanism. Thorax.

[25] Crothers K, Butt AA, Gibert CL, Rodriguez-Barradas MC, Crystal S, Justice AC, Veterans Aging Cohort 5 Project Team (2006). Increased COPD among HIV-positive compared to HIV-negative veterans. Chest.

[26] Diaz PT, King MA, Pacht ER, Wewers MD, Gadek JE, Nagaraja HN (2000). Increased susceptibility to pulmonary emphysema among HIV-seropositive smokers. Ann Intern Med.

[27] Sethi S, Muscarella K, Evans N, Klingman KL, Grant BJ, Murphy TF (2000). Airway inflammation and etiology of acute exacerbations of chronic bronchitis. Chest.

[28] Alan Lifson R, Jacqueline Neuhaus, Jose Ramon Arribas, Mary van den Berg-Wolf, Ann Labriola M, Timothy RH, the INSIGHT SMART Study Group (2010). Smoking-related health risks among persons with HIV in the strategies for management of antiretroviral therapy clinical trial. Am J Pub Health.

[29] Helleberg M, Shoaib Afzal, Gitte Kronborg, Carsten Larsen S, Gitte Pedersen, Court Pedersen (2013). Mortality attributable to smoking among HIV-1-infected individuals: a nation wide, population-based cohort study. CID.

